# Mindful Aging: The Effects of Regular Brief Mindfulness Practice on Electrophysiological Markers of Cognitive and Affective Processing in Older Adults

**DOI:** 10.1007/s12671-015-0482-8

**Published:** 2015-12-28

**Authors:** Peter Malinowski, Adam W. Moore, Bethan R. Mead, Thomas Gruber

**Affiliations:** 1 Research Centre for Brain and Behaviour, Liverpool John Moores University, Liverpool, L3 3AF UK; 2 Department of Psychological Sciences, Institute of Psychology, Health and Society, University of Liverpool, Liverpool, UK; 3 Institute for Psychology, University of Osnabrück, Osnabrück, Germany

**Keywords:** Cognitive aging, Mindfulness meditation training, Executive control, Emotion regulation, Event-related potentials, Randomized active controlled study

## Abstract

There is growing interest in the potential benefits of mindfulness meditation practices in terms of counteracting some of the cognitive effects associated with aging. Pursuing this question, the aim of the present study was to investigate the influence of mindfulness training on executive control and emotion regulation in older adults, by means of studying behavioral and electrophysiological changes. Participants, 55 to 75 years of age, were randomly allocated to an 8-week mindful breath awareness training group or an active control group engaging in brain training exercises. Before and after the training period, participants completed an emotional-counting Stroop task, designed to measure attentional control and emotion regulation processes. Concurrently, their brain activity was measured by means of 64-channel electroencephalography. The results show that engaging in just over 10 min of mindfulness practice five times per week resulted in significant improvements in behavioral (response latency) and electrophysiological (N2 event-related potential) measures related to general task performance. Analyses of the underlying cortical sources (Variable Resolution Electromagnetic Tomography, VARETA) indicate that this N2-related effect is primarily associated with changes in the right angular gyrus and other areas of the dorsal attention network. However, the study did not find the expected specific improvements in executive control and emotion regulation, which may be due to the training instructions or the relative brevity of the intervention. Overall, the results indicate that engaging in mindfulness meditation training improves the maintenance of goal-directed visuospatial attention and may be a useful strategy for counteracting cognitive decline associated with aging.

**Electronic supplementary material:**

The online version of this article (doi:10.1007/s12671-015-0482-8) contains supplementary material, which is available to authorized users.

## Introduction

In recent years, interest in the psychological and neural effects of mindfulness meditation practice has grown significantly and emerging evidence demonstrates positive effects on cognitive (Chiesa et al. 2011; Malinowski 2013b; Slagter et al. 2011) and emotional (Goldin and Gross 2010) processes. Furthermore, a range of different mindfulness-based interventions (Chiesa and Malinowski 2011) have shown benefits for the treatment of several clinical problems such as anxiety disorders and depression (Hofmann et al. 2010; Piet and Hougaard 2011), chronic pain (Veehof et al. 2011), and substance-use disorders (Chiesa and Serretti 2014), as well as improving well-being and behavioral regulation (Keng et al. 2011). A recent comprehensive meta-analysis concludes that mindfulness-based therapies constitute an effective treatment for a variety of psychological problems and are particularly effective in reducing depression, anxiety and stress-related problems (Khoury et al. 2013).

Encouraged by previous findings with different adult populations and some further, largely indirect, indications (Lazar et al. 2005; Pagnoni and Cekic 2007; van Leeuwen et al. 2009), it has been suggested that mindfulness practices may also be beneficial in counteracting some of the physiological (Epel et al. 2009; Epel et al. 2013; Jacobs et al. 2011) and cognitive (Kaszniak 2011) effects associated with aging, and first programs have been proposed (Zellner Keller et al. 2014).

A closer look at existing evidence reveals extensive gaps. First of all, the understanding of *specific* improvements in cognitive or emotional processing even in younger adult populations is still limited (Malinowski 2013b). The vast majority of available data stem from investigating the effects of standard interventions such as mindfulness-based stress reduction (MBSR), mindfulness-based cognitive therapy (MBCT) and similar programs. As these programs include a whole range of different exercises and activities, only some of which involve mindfulness meditation practice, they do not offer the required specificity for determining which components are responsible for observed changes. Hence, when studying these comprehensive interventions it is not possible to attribute their established beneficial outcomes unequivocally to mindfulness practices, as opposed to other components within the program.

While for clinical purposes establishing overall effectiveness of these complex programs may suffice, it is inadequate for unravelling the psychological, physiological and neural mechanisms underlying the observed changes. To achieve this, a more fine-grained approach that studies the effects of *individual program components* as well as their interactions will be required. Generating such understanding is of even greater importance when considering that the field of mindfulness-based programs is moving towards diversification, for example, in terms of time frames of the interventions (e.g., Ding et al. 2014; Tang et al. 2014), delivery mode (e.g., Davis and Zautra 2013; Krusche et al. 2012; Thompson et al. 2010; Zautra et al. 2012), or populations and conditions that are being targeted (e.g., Reb et al. 2012; Zenner et al. 2014). To inform the choices that are made when such new developments take place, a solid understanding as to whether the different program components exert any influence, what their influence is, and how it contributes to positive outcomes will be paramount. Although the first steps in this direction have been made by investigating the effects of a simple mindful breath awareness practice (Moore et al. 2012) and of body scan practices (Ditto et al. 2006; Mirams et al. 2013); overall, this research is still in its infancy.

An even larger gap exists regarding the questions of whether mindfulness meditation may be beneficial for older adults in terms of improving their cognitive processing and whether it may even contribute to preventing age-related cognitive decline. Recent reviews by Gard et al. (2014) and Luders (2014) considered related evidence regarding age-related cognitive decline and neural degeneration, respectively. Both reviews demonstrate that although there are a few positive indicators, so far evidence is too limited and varied and more rigorous research is required. Given that the research into mindfulness and aging primarily utilized multi-component programs, the necessity of investigating specific mindfulness components also in this context is evident.

In relation to psychological interventions, mindfulness is usually defined as a mental state characterized by “paying attention on purpose, in the present moment, and nonjudgmentally to the unfolding of experience moment by moment” (Kabat-Zinn 2003). It is thought that the ability to be mindful can be enhanced by refining ones attentional skills and developing an open, non-evaluative attitude toward the different mental experiences that may arise (Bishop et al. 2004; Malinowski 2008).

Different theoretical frameworks which outline how mindfulness practices exert their beneficial influence agree on the notion that attention is a core function to be developed (Hölzel et al. 2011; Lutz et al. 2008; Malinowski 2013b; Shapiro et al. 2006; Slagter et al. 2011; Wallace and Shapiro 2006). In line with this, a range of studies indicate that mindfulness practices improve several attentional functions such as (i) the ability to sustain attention over time (Moore and Malinowski 2009; Pagnoni et al. 2008; Valentine and Sweet 1999), (ii) the allocation of attentional resources (Slagter et al. 2007; Slagter et al. 2009), and (iii) attentional control functions, in particular the ability to withhold or inhibit automatic responses (Manna et al. 2010; Moore et al. 2012; Moore and Malinowski 2009).

Recently, Malinowski (2013b) outlined how different attentional functions and underpinning attentional networks map onto the main phases of mindful breath awareness practice, a form of meditation that requires maintaining sustained attention to somatic sensations associated with inhaling and exhaling. The key to this process is the monitoring and regulation of one’s attentional state, achieved by the interplay of the salience network (Dosenbach et al. 2007; Dosenbach et al. 2006; Seeley et al. 2007; Sridharan et al. 2008), the executive control network, and the orienting network (Posner 2012; Posner and Rothbart 2007). It is worth noting here that the observed positive effects on cognitive functions appear to propagate to modalities different from the meditation practice itself, i.e., from the domains of somatosensation and the awareness of own thoughts and feelings during the practice to the visual (Moore et al. 2012) and the auditory (Antonova et al. 2015; Cahn and Polich 2009) domain and performance on a range of cognitive tasks (Chiesa et al. 2011).

Refined attention skills as those described above are thought to underpin emotional and cognitive flexibility and a more accepting mental attitude, leading to improved well-being (Chambers et al. 2009; Malinowski 2013a, 2013b). In particular, Teper et al. (2013) proposed that improvements in attentional control would lead to improved emotion regulation processes. As heightened present-moment awareness provides better access to internal cues indicating that control is required, it in turn leads to more effective control and emotion regulation. Indeed, a recent study demonstrated that 16 weeks of brief, regular mindful breath awareness practice lead to commensurate changes in neural activity during the Stroop task—a canonical measure of executive control. Enhanced neural activity indicative of improved selective attention to relevant stimulus features was followed by more efficient response conflict resolution, indicated by reduced neural resource demands (Moore et al. 2012).

In addition, Teper and Inzlicht (2013) provide evidence that the mindful acceptance of internal mental states is integral to effective executive control processes and participation in a 3-month intensive mindfulness meditation retreat concurrently resulted in enhanced response inhibition performance and improved socio-emotional functioning (Sahdra et al. 2011). Using an emotional Stroop task Allen et al. (2012) found that after six weeks of meditation training the efficiency of conflict resolution improved in the meditation group but not in the active control group. These improvements were associated with increased activation of the dorsolateral prefrontal cortex (DLPFC) during task execution. As the DLPFC is part of the executive control network (Raz and Buhle 2006; Seeley et al. 2007) this finding is likely to represent improvements in attentional control. Furthermore, the total time participants had invested in meditation practice predicted increased activity in the anterior insula and the cingulate cortex, both of which are part of the salience network (Buckner et al. 2008; Seeley et al. 2007). Investigating the processing of very briefly presented and subsequently masked emotional scenes in experienced meditators compared to matched non-meditators, Nielsen and Kaszniak (2006) found evidence for enhanced emotion regulation.

Although still limited, the reviewed findings are generally in line with the hypothesis that regular mindfulness meditation practice enhances emotion regulation, improvements which might be based on a progression from enhanced attentional control and the involvement of the executive control network, to improved emotion regulation skills, indexed by the selective involvement of the salience network (Malinowski 2013b).

In light of the extant evidence and related theoretical considerations we would expect that also when older adults engage in mindfulness practice improvements in executive control and emotion regulation will ensue. As cognitive decline is of serious concern in the aging population (Salthouse 2004, 2009; Tucker-Drob 2011) and a range of studies demonstrate the prevalence of executive deficits in older adults (Andrés et al. 2008; Cohn et al. 1984; Ludwig et al. 2010; Mayas et al. 2012; Panek et al. 1984; Van der Elst et al. 2006; West and Alain 2000; West and Bell 1997; Zysset et al. 2007), executive control improvements would indicate the potential of meditation practice to protect against age-related decline. Although psychological well-being and emotion regulation skills appear to generally increase in older adults (Carstensen et al. 2000; Charles and Carstensen 2010; Nilsson et al. 2010), due to the fact that many older adults are exposed to ongoing stressful situations, for example caring for a chronically ill family member, improvements in emotion regulation skills would nevertheless be relevant (Nielsen and Kaszniak 2006).

**Fig. 1:**
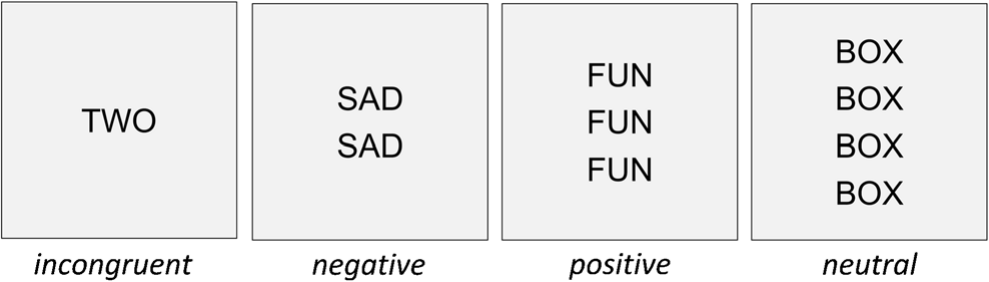
Stimulus examples from each semantic category (incongruent, negative, positive, neutral), and each list size (1–4 words). This presentation is not to scale and word sizes relative to screen size are exaggerated

Most of the studies considered above investigate executive control and emotion regulation skills separately. However, the “emotional-counting Stroop task” (ecStroop), introduced by Whalen et al. (1998), combines two well-established versions of the Stroop task, the “counting Stroop task” (cStroop; Bush et al. 1998) and the “emotional Stroop task” (eStroop; J. M. G. Williams et al. 1996). In the ecStroop task participants have to indicate by button press how many words are presented on the screen (between one and four). Executive control is required in the response conflict condition, i.e., when the meaning of a presented number word is at odds with the number of words that are presented and need to be responded to (e.g., “TWO” written three times; answer: “three”). Emotion regulation is involved when different affective states are induced by including words that are emotionally neutral (e.g., “DOOR”), positive (e.g., “LOVE”) or negative (e.g., “PAIN”), as participants have to shield themselves from the emotion that has been triggered by the automatic processing of the word meaning (see Fig. 1 for stimulus examples).

fMRI evidence indicates that the affective subdivision of the anterior cingulate cortex (ACC) is involved in this process, as this brain area was activated in an ecStroop task by stimuli of negative valence only (Whalen et al. 1998) in. Similar emotional Stroop paradigms are frequently used in patient samples. When patients are presented with color words relevant to their current concerns or condition the automatic processing of the word meaning delays naming of the word’s color (J. M. G. Williams et al. 1996). Such increases in response latency have been found for general anxiety disorder (Mathews and MacLeod 1985), phobias (Watts et al. 1986), social phobia (Hope et al. 1990), panic disorder (McNally et al. 1992), post-traumatic stress disorder (McNally et al. 1990), and obsessive-compulsive disorder (Foa et al. 1993). However, in healthy adults, emotional stimuli usually do not increase response latencies (J. M. G. Williams et al. 1996).

The aim of the present study was to investigate the effects of mindfulness training (MT) on executive control and emotion regulation in older adults. To achieve this within one task, we employed the ecStroop task, using behavioral measures (response times, accuracy) in conjunction with electrophysiological measures (event-related potentials; ERPs) of underlying neural processes. We expected to find MT-related improvements in electrophysiological measures of executive control and emotion regulation. Improvements in behavioral measures were only expected for executive control, because in healthy adults behavioral responses are usually not affected by emotion manipulations Stroop word tasks. To control for a wide range of extraneous variables including group contact time, daily exercise time, experimenter contact, group allocation, learning new information, participants’ intention and motivation, and exercise environment a matched active control group that involves active, effortful cognitive processes was implemented. We expected to find improvements in executive control and emotional processing in the mindfulness meditation group above and beyond any changes found in the active control group.

## Method

### Participants

Fifty-six older adults (range: 55–75 years; mean: 64.5 years; 15 males) were recruited from the general population (retired: *N* = 38; working: *N* = 18). The sample had a varied educational background (ranging from no formal qualification to postgraduate degrees), with a mean of 13.0 years spent in education. All participants provided written, informed consent. The study was carried out in line with the ethics guidelines of the British Psychological Society and was approved by the Research Ethics Committee at Liverpool John Moores University. Participants were randomly allocated to the mindfulness training group or to the active control group. Upon completion of the study all participants were reimbursed with £40 worth of high street shopping vouchers.

### Procedure

Participants in the mindfulness training group (MTG) were introduced to a simple, mindful breath awareness meditation taught by a meditation teacher of a Tibetan Buddhist tradition with close to 20 years of teaching experience. Participants were required to focus their attention on the sensations accompanying their breathing, either attending to the experience at the nostrils, around the diaphragm or the movement of the abdomen when inhaling and exhaling, without manipulating the breath in any form. Whenever attention would slip or wander off, the task would be to become aware of it and, without further elaboration, to redirect the focus of attention back to the sensation of breathing. Participants were instructed to recognize other arising thoughts, feelings or sensations, trying not to judge or evaluate them, and maintain a curious, non-elaborating attitude toward them.

This meditation instruction is in line with common psychological mindfulness conceptualizations that emphasize the development of attentional abilities combined with a specific, non-judgmental, and non-evaluative attitude toward the different mental experiences that may arise (Bishop 2002; Chiesa and Malinowski 2011; Malinowski 2008; Shapiro et al. 2006). The involvement of the different attentional functions is considered in detail in Malinowski (2013b). For the period of 8 weeks, participants were asked to meditate regularly for a minimum of 10 min per day, at least 5 days per week. Over the course of the 8 weeks, they attended four 90-min group sessions that consisted of psycho-educative components, inquiry-based discussions, and group meditation practice. The participants did not receive any particular instructions regarding body posture beyond the emphasis of trying to sit in an upright, relaxed position with a straight back.

To match the MT condition as closely as possible, participants allocated to the active control condition took part in a brain training group (BTG). The training involved active, effortful cognitive processes, rather than relaxation training, which does not mimic the assumed active “ingredients” of mindfulness practice well enough and has previously been shown to not fully account for the non-specific effects of MT (Ortner et al. 2007; Polak 2009; Tang et al. 2007). Participants in the BTG were asked to engage in mental arithmetic calculations, which involve effortful cognitive processing and activate a wide range of frontal and parietal brain regions implicated in attention (Fehr et al. 2007; Kong et al. 2005; Rickard et al. 2000). Additionally, such exercises are included in various commercially available brain training (BT) packages that claim to improve cognitive performance in older adults (e.g., Kawashima 2008).

The group sessions matched the MTG for frequency (four sessions) and duration (6 h total) and included psycho-educative components in form of healthy aging lectures (e.g., brain changes related to aging, nutrition, lifestyle choices, etc.), group discussions, and in-group exercises. Participants in the BTG were given exercise booklets to complete at home. The booklets contained 100 arithmetic calculations (approximately 10–15 min of practice) to be completed 5 days per week, for 8 weeks, plus additional calculations that the participant could complete at their leisure. As with MT, participants were instructed to complete the daily exercises while sitting upright in a quiet area. Thus, the active control condition also included elements of learning, discussion, and active practice (BT exercises) and controlled for a wide range of extraneous variables including group contact time, group session dynamics, daily exercise time, experimenter contact and motivation, learning new information, participants’ intention and motivation, and exercise environment. Interestingly, several participants engaged more with the BT than requested, indicating that also in terms of motivation and outcome expectations the groups were comparable.

Before being allocated to the specific training group and attending the first group session (i.e., at baseline/T1) and after completion of the training program (at T2) all participants completed several questionnaires and engaged in a set of computer-based tasks while their EEG was recorded. Here we only report the results from the ecStroop task—further results will be published in the near future.

Participants completed the ecStroop task to assess changes to executive control functions and to the assumed involvement of emotional processing. In each trial of the task a stimulus consisting of one to four (list sizes 1 to 4) identical and centrally aligned words was presented. Stimuli consisted of 28 English language words spread across four semantic categories as follows: incongruent, negative, positive, and neutral. For list size 1, the word was presented centrally on the screen. For list size 2, two words were presented 0.6 degrees of visual angle above and below the center of the screen. For list size 3, one word was presented centrally and two words presented 1.2 degrees of visual angle above and below the center of the screen, respectively. For list size 4, two words were presented 0.6 degrees above or below the center and two words presented 1.8 degrees above or below the center. Stimuli were presented in black (Arial, font size 48) on a light gray background. Figure 1 presents one example stimulus from each list size and semantic category.

The participant’s task was to indicate the number of words that appeared on the screen, ignoring the word’s meaning. Four response keys were used to enter responses; these were labelled with the numbers 1–4 for the responses 1 to 4, respectively. Only four number words (ONE to FOUR) were chosen in the incongruent condition to limit the number of potential responses for the task. Each of the other three conditions contained eight potential words in order to minimize exposure effects. Each incongruent stimulus could be presented in the three list sizes that do not match its meaning (e.g., list size 1, 2, and 4 for the number word “THREE”), while all other stimuli could be presented in all four list sizes.

The mean frequency of use in English language (Leech et al. 2001) was matched across the negative, positive, and neutral category [F(2, 21) = 0.002, *p* = 0.998]. It was neither possible nor necessary to achieve a frequency match also for the incongruent category, as number words are some of the most frequently used words in the English language, which is an essential aspect of the interference-related task manipulation. In the incongruent condition, participants must ignore the meaning of the number word in order to respond accurately to the number of words presented. Therefore, the high frequency of use and thus, familiarity in the incongruent condition is essential as it drives the conflict element of the task. The stimuli were matched for word length across all four conditions.

Each trial began with a fixation cross presented centrally for 500 ms, which was replaced by a word stimulus (list size 1 to 4) for 1500 ms. The trial ended with a variable inter-trial interval of between 850 and 1100 ms, during which the fixation cross was presented again. The complete task consisted of four blocks of 63 randomly intermixed trials (252 total), separated by 20-s breaks. The incongruent condition accounted for 60 trials (4 words × 3 list sizes × 5 repeats) while the remaining 3 conditions accounted for 64 trials each (8 words × 4 list sizes × 2 repeats).

### Measures

MTG and BTG both completed brief weekly training logs, recording how many days they had engaged with the training that week and the amount of time they had spent on average each day completing the exercises. The respective diaries were not intended to produce data that could be used for a between groups statistical comparison of adherence or to assess dose related effects on behavioral or electrophysiological markers of performance. They did, however, provide adequate data to ascertain if the interventions had been followed as instructed. Further, we opted for a light-touch approach to minimize the burden of additional paperwork.

As a general slowing in processing speed has previously been proposed as a cause for age-related cognitive declines (Salthouse 2000), a very short and simple response-time task was administered at T1 to confirm that speed of processing was comparable between groups. The task was set up as follows: A white dot was displayed in the middle of a computer screen a total of 20 times, with the participant having to press a button as quickly as possible in response to seeing the dot. The first 5 trials were ignored with the final 15 trials averaged to give a mean reaction time for each participant that could be utilized to approximate speed of processing.

It is well-established that human aging is associated with declines in working memory function (Craik and Salthouse 2000) and some evidence suggests that mindfulness training improves working memory (Chambers et al. 2008; Jha et al. 2010). Given the close link between attention and working memory (see Gazzaley 2011 for a recent review), it was important to control for working memory capacity between groups so that any observed improvements in performance may be attributed to modulations of attentional functions and the neural mechanisms that subserve them, rather than to a pre-existing deficit or modulation of working memory. To limit participant burden, we incorporated a single task from a pre-existing working memory test battery. The Spatial Short Term Memory task (SSTM; Lewandowsky et al. 2010) was chosen as it has been shown to have high loadings on working memory capacity factors and is highly correlated with measures of reasoning and general fluid intelligence (Lewandowsky et al. 2010; Oberauer 2005; Oberauer and Süß 2000; Oberauer et al. 2003).

WM tasks involving digit and operational span were specifically avoided due to a conflict with the arithmetic calculations included in the BTG training. In short, the SSTM consists of trials wherein 1 to 6 dots are consecutively displayed into cells of a 10 × 10 grid, with only one dot appearing on the screen at a time. Participants are instructed to remember the spatial relations between dots and to then reproduce the overall pattern of dots, using a standard mouse, into a blank grid following a brief mask at the end of the stimulus presentation. The dependent variable, SSTM total score, is calculated based on points awarded for how closely the participant reproduces the overall pattern (2 points awarded for reproducing a dot exactly and 1 point for a deviation of one cell in any direction). The full set up and calculation of scores for the SSTM are described in detail by Lewandowsky et al. (2010).

Until now, no valid questionnaire for capturing longitudinal changes in mindfulness has been established. Nevertheless, we decided to include the Five Facet Mindfulness Questionnaire (FFMQ; Baer et al. 2006; Baer et al. 2008) to gain some indication whether self-reported mindfulness was influenced by the training. The FFMQ has a five-factor structure: (1) *Non-reactivity to inner experience* (FFMQ-NR), (2) *Observing* internal and external sensations including thoughts, emotions, sights, sounds, and smells (FFMQ-O), (3) *Acting with awareness* describes attending to one’s actions in the present moment and can be contrasted with automatic, impulsive, or habitual behaving (FFMQ-A), (4) *Describing* involves labelling internal experiences with words (FFMQ-D), and (5) *Non-judging of experience* means refraining from value judgments or self-criticism (FFMQ-NJ). In addition to analyzing the five factors also a total mindfulness score is calculated by summing up all items (range: 39–195). Internal consistencies (Cronbach alpha) between 0.75 and 0.91 have been reported for these facets (Baer et al. 2006).

To account for the possibility that observed between-group differences merely result from changes in self-efficacy, mental well-being or cognitive and physical activity we included several control measures. Increases in self-efficacy may feasibly be gained from positively attempting to address cognitive decline via enrolment in the study. We thus included the Generalized Self-Efficacy Scale (GSE; Schwarzer and Jerusalem 1995) pre and post-training. The GSE is a 10 item scale designed to assess a general sense of perceived self-efficacy, with a possible score range of 10 to 40. Higher scores indicate stronger perceived self-efficacy. The GSE has a high reliability and construct validity (Leganger et al. 2000; Schwarzer et al. 1999) and Cronbach alpha ranges from 0.75 to 0.94 across a number of different language versions (Luszczynska et al. 2005).

Mental well-being was measured using the Warwick-Edinburgh Mental Well-Being Scale (WEMWBS; Tennant et al. 2007). The scale contains 14 positively worded items with scores ranging from 14 to 70. Confirmatory factor analysis has supported the single factor structure and internal consistency (alpha = 0.91) and test-retest reliability (0.83) are high (Tennant et al. 2007).

Levels of cognitive and physical activity were measured using an adapted version of a widely used scale (Verghese et al. 2003), which was devised for use in a retired population. As such, the items only account for leisure activities that are typically carried out in free time. As retired individuals inherently have more free time than working peers, the scale is biased towards retired individuals reporting a greater cognitive and physical activity, which is intrinsically untrue as the majority of jobs contain both cognitive and physical elements. As our sample included both retired and working individuals, the original scale was unsuitable. We thus added 12 items to provide a more comprehensive assessment of current/ongoing cognitive and physical activity. This revised scale included 29 items in total, 14 related to cognitive activities and 15 related to physical activities. Participants reported frequency of participation as “daily,” “several days per week,” “once weekly,” “monthly,” “occasionally,” or “never.” Responses were coded to generate an overall cognitive-activity score, ranging from 0 to 98, and a physical-activity score, ranging from 0 to 105. This revised scale has not been tested previously and is here merely used to provide a broad estimate of current/ongoing cognitive and physical activity and to ensure that the two groups did not differ significantly in this respect.

### Data Analyses

During task completion EEG was recorded continuously from 64 active Ag/AgCl electrodes with a BioSemi Active-Two amplifier system (BioSemi, Amsterdam, Netherlands). For monitoring eye movements and blinks the horizontal and vertical electrooculogram (EOG) was recorded with supra-and infraorbital electrodes on the left eye and two electrodes placed next to the external canthi. EEG and EOG were sampled at 512 Hz. Two additional electrodes (Common Mode Sense [CMS] and Driven Right Leg [DRL]) were used as reference and ground (see www.biosemi.com/faq/cms&drl.htm for details). EEG was segmented to obtain epochs starting 200 ms prior and 800 ms following stimulus onset. Pre-processing of data was performed using EEGLAB (Delorme and Makeig 2004) and the ERPLAB (Lopez-Calderon and Luck 2014) and FASTER (Nolan et al. 2010) plugins, the latter for artifact removal. Based on a predefined z-score threshold of ±3 for each parameter, artifacts were detected and corrected regarding single channels, epochs, independent components (based on the infomax algorithm; Bell and Sejnowski 1995), and single-channel single-epochs. Remaining artifactual independent components and epochs containing artifacts were removed after visual inspection. Data were filtered offline with a 1-Hz high pass filter. Prior to the independent component analysis a pre-stimulus baseline from −100 to 0 ms was applied. Fz was used as reference during pre-processing and data were subsequently algebraically re-referenced to average reference.

To analyze which brain areas are associated with observed meditation-specific changes, we applied Variable Resolution Electromagnetic Tomography (VARETA; Bosch-Bayard et al. 2001) to localize the cortical generators of those ERP components that were selectively modulated by meditation practice. The VARETA approach provides the spatially smoothest intracranial distribution of current densities in source space which is most compatible with the amplitude distribution in electrode space (Gruber et al. 2006). The inverse solution consisted of 3244 grid points (“voxels”) of a 3D-grid (7 mm grid spacing). This grid and the arrangement of 64 electrodes were placed in registration with the average probabilistic MRI brain atlas (“average brain”) produced by the Montreal Neurological Institute (MNI; Evans et al. 1993). Activation threshold correction to account for spatial dependencies between voxels was calculated by means of Random Field Theory (Worsley et al. 1996). To verify the reliability of this source localization approach for the current data set the origins of the occipital P1 ERP component (100–120 ms post-stimulus) were calculated. As detailed in Online Resource 1 the localization of the cortical sources in the lateral occipitotemporal areas corresponds to the P1 sources consistently reported in the literature (e.g., Di Russo et al. 2002; Proverbio and Adorni 2009), thus confirming that the chosen localization approach accurately detects underlying cortical sources.

Individual data sets with more than 30 % loss of data were excluded from further analysis. This was primarily due to the excessive movement of artifacts, likely resulting from the fact that a relatively large number of participants had limited or no prior experience with using computers. Furthermore, based on our a priori criterion participants were excluded from both electrophysiological and behavioral analyses if they had a hit rate below 85 % in the neutral condition, as this would suggest either poor task understanding or/and difficulties with response-key mapping. After applying these criteria data were available for 22 participants in each group for behavioral analyses and 18 participants in each group for ERP analyses (MTG: 3 males/15 females; BTG: 4 males/14 females). For each time point *t* tests confirmed that the amount of available data was comparable across groups (*p* > 0.05). The pattern of behavioral results was similar when all available data or only the data of participants included in the ERP analyses were used. Thus, the behavioral analyses are presented for all available data.

## Results

### Test for Pre-Training Differences Between Groups

To confirm that the randomization procedure was successful and the two groups did not differ on important variables a range of *t* tests were calculated for all participants included in the final statistical analyses. This analysis confirmed that the groups are comparable in terms of age, dispositional mindfulness, computer ability, years in education, health, speed of processing, working memory capacity (SSTM), self-efficacy, mental well-being, and ongoing/current cognitive and physical activity (all *p* > 0.05; see Online Resource 2 for details). Control over so many extraneous variables ensures that between group differences can be strongly interpreted as a consequence of the cognitive training interventions. In addition, we considered each of the four stimulus categories of the ecStroop and confirmed that there were no significant between-group baseline differences for response times (all *p* > 0.66), accuracy (all *p* > 0.06) and for any of the analyzed ERP components (all *p* > 0.57).

As a task manipulation check, pooled T1 (*N* = 50, also including participants without T2 data) response time (RT) means and hit rates (HRs) were subjected to separate one-way within-participant ANOVAs. These ANOVAs yielded significant main effects for RTs [F(1.95, 95.85) = 82.238, *p* < 0.001] and HRs [F(1.42, 69.62) = 14.493, *p* < 0.001]. Post hoc pairwise comparisons clearly demonstrated that the incongruent condition produced slower RTs (*p* < 0.001; Bonferroni adjusted) and lower HRs (*p* < 0.005; Bonferroni adjusted) than the other three conditions, confirming that the conflict introduced in the incongruent condition increased processing requirements. The manipulation of affective valence (neutral, positive, negative) neither influenced RTs (*p* > 0.65) nor HRs (*p* > 0.27), confirming previous observations that in healthy participants the manipulation of emotional valance is typically not reflected in behavioral performance.

In general, the participants in both groups managed to adhere to the required exercise schedule. Based on the training logs, the approximate time spent completing daily exercises was 13 min for the MTG and 11 min for the BTG. On average, both the MTG and BTG engaged on 5 days per week with their exercises.

### Results from Control Variables

The total mindfulness score (FFMQ) and the scores for each of the five FFMQ subscales were subjected to separate *Time* (2) × *Group* (2) mixed ANOVAs. For the total mindfulness score no significant main or interaction effects were found. Analysis of the FFMQ subscales revealed a significant *Group* × *Time* interaction [F(1, 48) = 15.907, *p* < 0.001, *r* = 0.499] for FFMQ-O with MTG significantly increasing FFMQ-O from T1 to T2 [*t*(24) = −3.642, *p* = 0.001], while there was a non-reliable trend for BTG to reduce FFMQ-O from T1 to T2 [*t*(24) = 1.945, *p* = 0.064]. A significant main effect of *Time* [F(1, 48) = 4.438, *p* = 0.040, *r* = 0.291] was found in FFMQ-A. Surprisingly, this effect was caused by a relative decrease in FFMQ-A from T1 to T2 (T1 = 27.4, T2 = 26.1). No other significant effects emerged from the analysis of the FFMQ subscales (see Online Resource 2 for further statistical details).

The self-efficacy scores (GSE) were subjected to a *Time* (2) × *Group* (2) mixed ANOVA, revealing a significant main effect of *Time* [F(1, 48) = 5.617, *p* = 0.022, *r* = 0.324] which indicated that overall mean scores increased slightly from T1 to T2 (T1 = 32.2, T2 = 33.2). This small albeit significant overall difference may illustrate that being a part of the study helped raise self-efficacy, potentially as the participants may have thought they were doing something positive by taking part. No significant between-group or interaction effects were found.

The WEMWBS mental well-being scores were subjected to a *Time* (2) × *Group* (2) mixed ANOVA, yielding no significant main or interaction effects. Submitting the SSTM working memory scores to a *Time* (2) × *Group* (2) mixed ANOVA revealed a non-significant *Group* × *Time* interaction [F(1, 45) = 1.322, *p* = 0.256], confirming that meditation-specific changes observed in this study are unlikely to result from modulations of working memory (see Online Resource 2 for further statistical details).

### Behavioral Results

RTs and HRs were subjected to *Time* (2) × *Condition* (4) × *Group* (2) mixed ANOVAs to determine intervention related changes in MTG and BTG. Significant main effects of *Time* were observed for RTs [F(1, 42) = 11.009, *p* = 0.002] and HRs [F(1, 42) = 5.907, *p* = 0.019], indicating that overall performance improved through participation in the training. Furthermore, there was a significant main effect of *Condition* for *RTs* [F(1.74, 73.22) = 126.523, *p* < 0.001] and *HRs* [F(1.29, 54.10) = 13.633, *p* < 0.001], reflecting that the incongruent condition had a detrimental effect on performance [pairwise comparisons all Bonferroni adjusted *p* < 0.001]. The remaining three conditions produced similar RTs.

For HRs a significant *Time* × *Condition* interaction [F(2.51, 105.53) = 4.542, *p* = 0.008] was also found. Paired *t* tests for each condition demonstrated that significant increases in accuracy occurred across groups for the incongruent [*t*(43) = −3.140, *p* = 0.003] and negative [*t*(43) = −2.087, *p* = 0.043] conditions but not for the positive [*t*(43) = −0.488, *p* = 0.628] or neutral [*t*(43) = −0.768, *p* = 0.447] conditions.

**Fig. 2:**
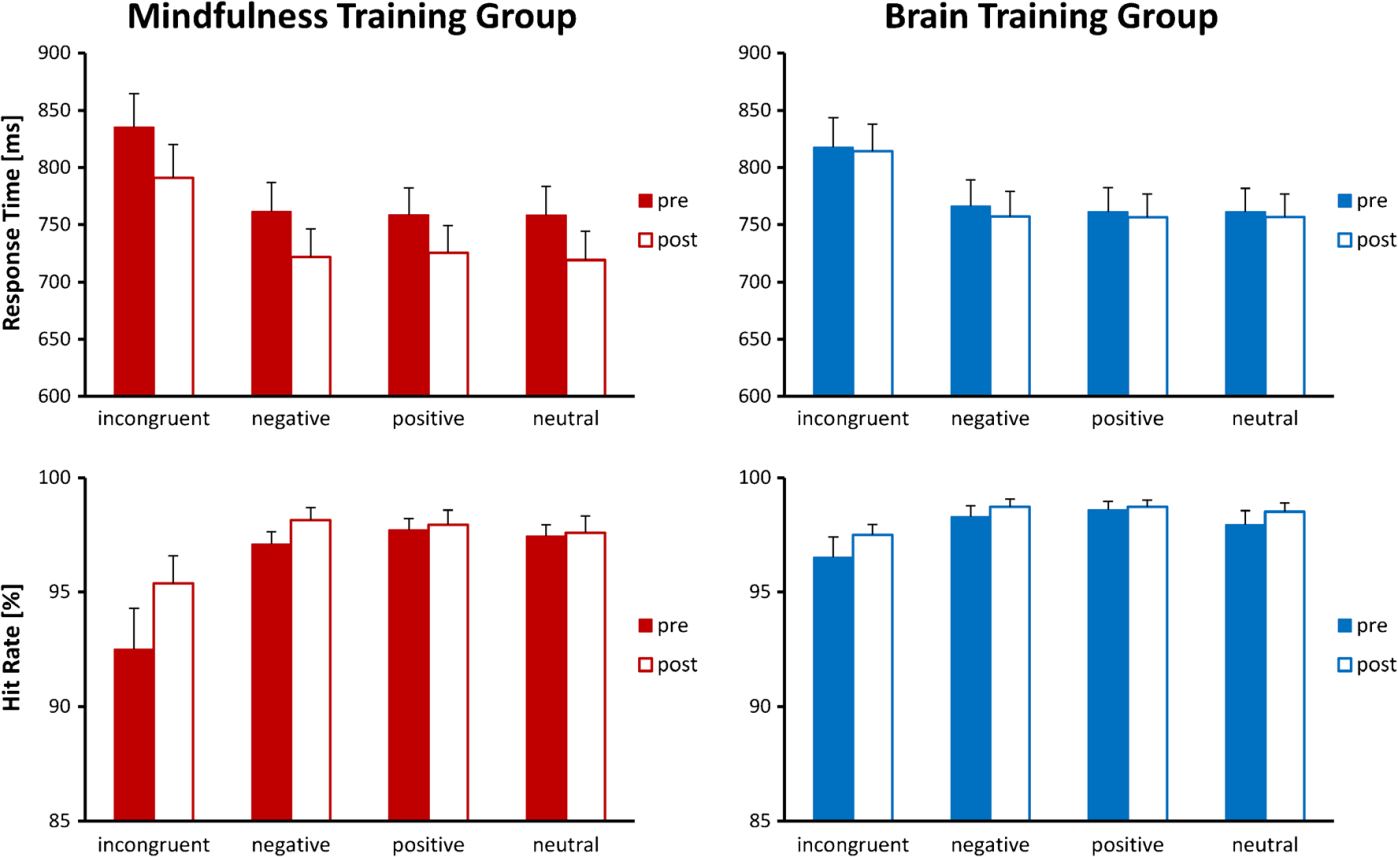
Behavioral performance for both groups and all four conditions before and after the training (*filled* and *unfilled bars*, respectively). *Error bars* indicate standard errors of the mean

Most importantly, a significant interaction between *Group* and *Time* emerged for RTs [F(1, 42) = 6.263, *p* = 0.016; *r* = 0.360], reflecting that RTs improved from T1 to T2 in the meditation group [T1: 776 ms, T2: 738 ms; *t*(21) = 4.407, *p* < 0.001], while for the BTG RTs did not change significantly [T1: 776 ms, T2: 770 ms; *t*(21) = 0.535, *p* = 0.598]. This interaction thus confirms a meditation-specific improvement in overall response times. See Figs. 2 and 6 for depictions of this effect and Online Resource 2 for further statistical details.

### Electrophysiological Results

The fronto-central N2 ERP component was best captured by a time window of 270 to 340 ms at electrode FCz. Correlating pooled RTs with mean N2 amplitude at FCz for T1 yielded a significant correlation [*r* = 0.304, *p* = 0.024, 1-tailed], reflecting that higher fronto-central N2 mean amplitudes are related to better performance (lower RTs) and confirming the importance of fronto-central N2 for task performance and justifying its position as the main focus of the ERP analysis.

**Fig. 3:**
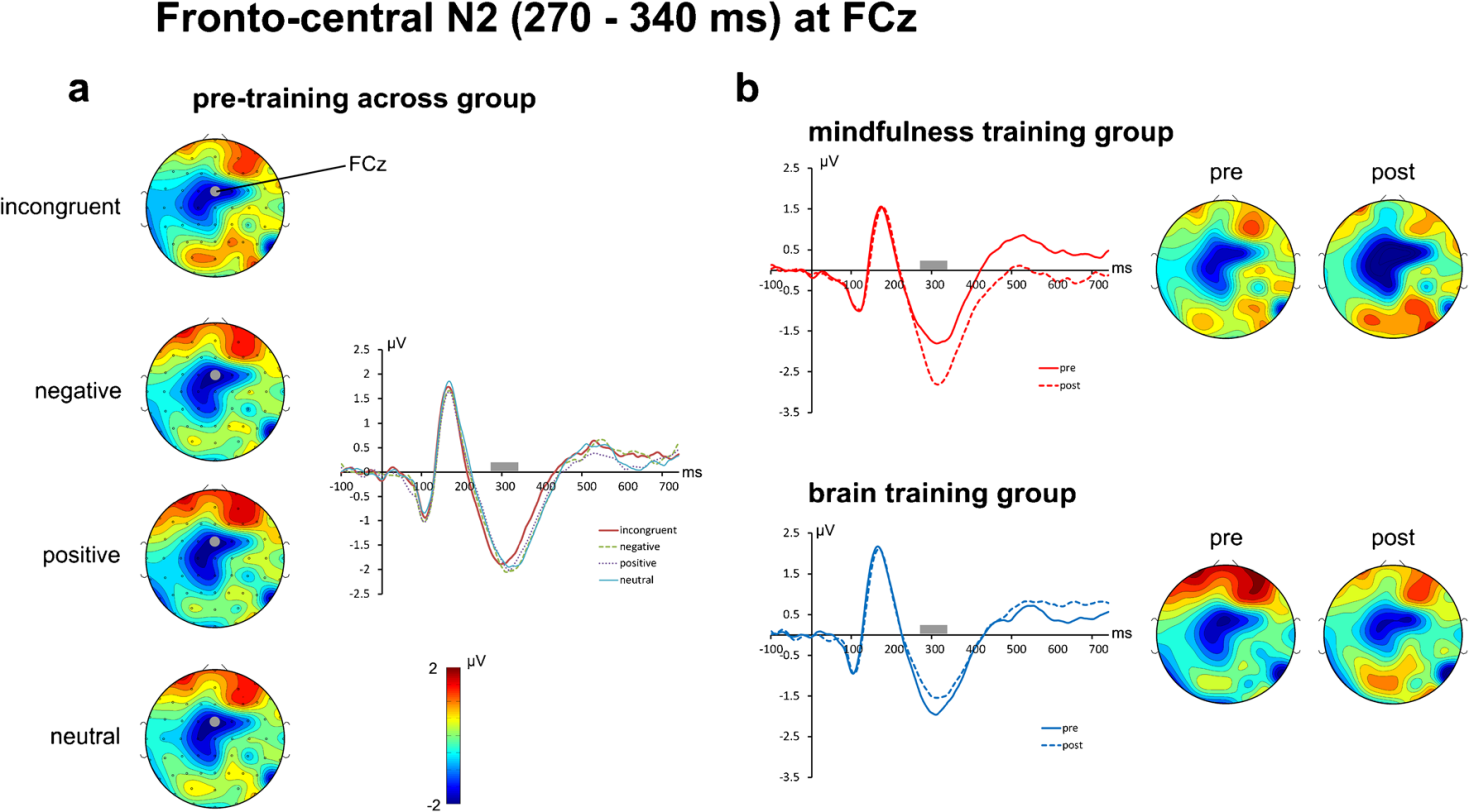
Event-related potentials (at electrode FCz) and spherical spline interpolated scalp topographies of the fronto-central N2 potential. The *left panel* (**a**) shows the scalp topographies for T1 (pre-training) averaged across meditation and brain training group. A clear N2 component captured in a time window from 270 to 340 ms post-stimulus (indicated by the *gray bars* in the ERP plots) and centered on electrode FCz is clearly visible. The *right panel* (**b**) shows the changes of the N2 from pre-training (*solid line*) to post-training (*dashed line*) for both groups

The fronto-central N2 mean amplitude was subjected to a *Time* (2) × *Condition* (4) × *Group* (2) mixed ANOVA revealing a significant *Group* × *Time* interaction [F(1, 34) = 6.989, *p* = 0.012; *r* = 0.413] and no further significant main effects or interactions (all *p* > 0.10). Paired samples *t* tests revealed a significant increase in the fronto-central N2 for the MTG across conditions (T1 = −1.72, T2 = −2.60; *t*(17) = 2.81, *p* = 0.012) whereas there were no significant changes for the BTG (T1 = −1.68, T2 = −1.50; *t*(17) = −0.75, *p* = 0.465). Figure 3 illustrates the differences in the ERP waveforms of each group, clearly demonstrating an increased N2 for MTG as compared to a relative (albeit not significant) decrease for BTG. Furthermore, changes in the N2 amplitude from T1 to T2 were positively correlated with reductions in RTs from T1 to T2 [*r* = 0.281, *p* = 0.049; pooled across groups].

**Fig. 4:**
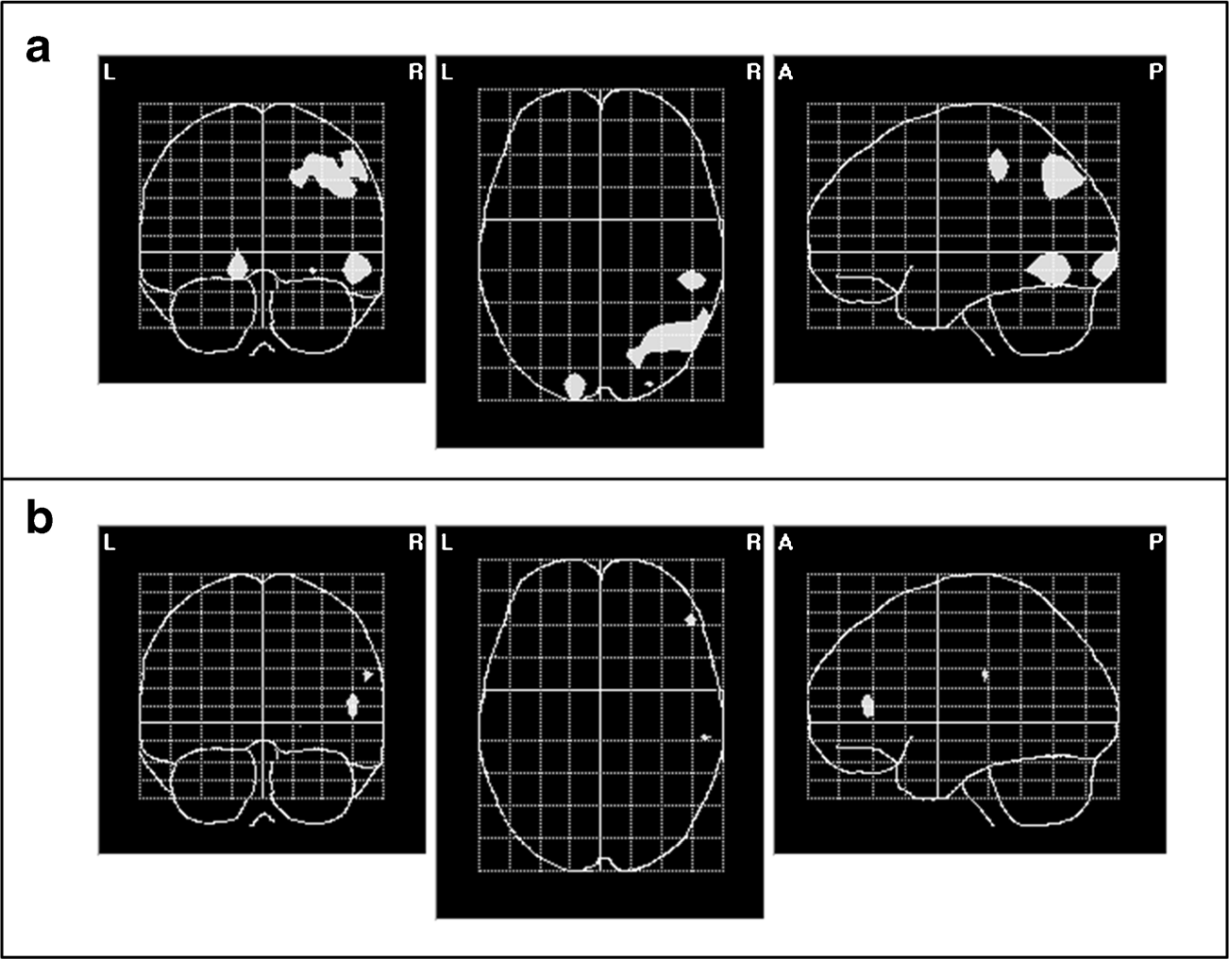
Inverse solution of the effect of time (T1 versus T2; Hotelling *T* ^2^ test; *p* < 0.05) of the N2 ERP component (270 to 340 ms post-stimulus onset) depicted in a glass brain. Statistically significant voxels are *printed in white. Upper panel* (**a**) solution for MTG. Centers of gravity: right angular gyrus (MNI coordinates: *X* = 50, *Y* = −33, *Z* = 48); right superior parietal lobe (MNI coordinates: *X* = 34, *Y* = −68, *Z* = 48); right inferior temporal lobe (MNI coordinates: *X* = 49, *Y* = −69, *Z* = −11) and left lingual gyrus (MNI coordinates: *X* = −3, *Y* = −91, *Z* = −9). The *lower panel* (**b**) shows the same for BTG, where only spurious voxels reach significance

Figure 4 depicts the neural sources associated with the N2-changes from T1 to T2. In line with the results from the ERP analysis, meaningful statistically significant changes were only observed for the MTG but not for the BTG. These changes were centered in the right angular gyrus, the right superior parietal lobe, the right inferior temporal lobe, and the left lingual gyrus.

**Fig. 5:**
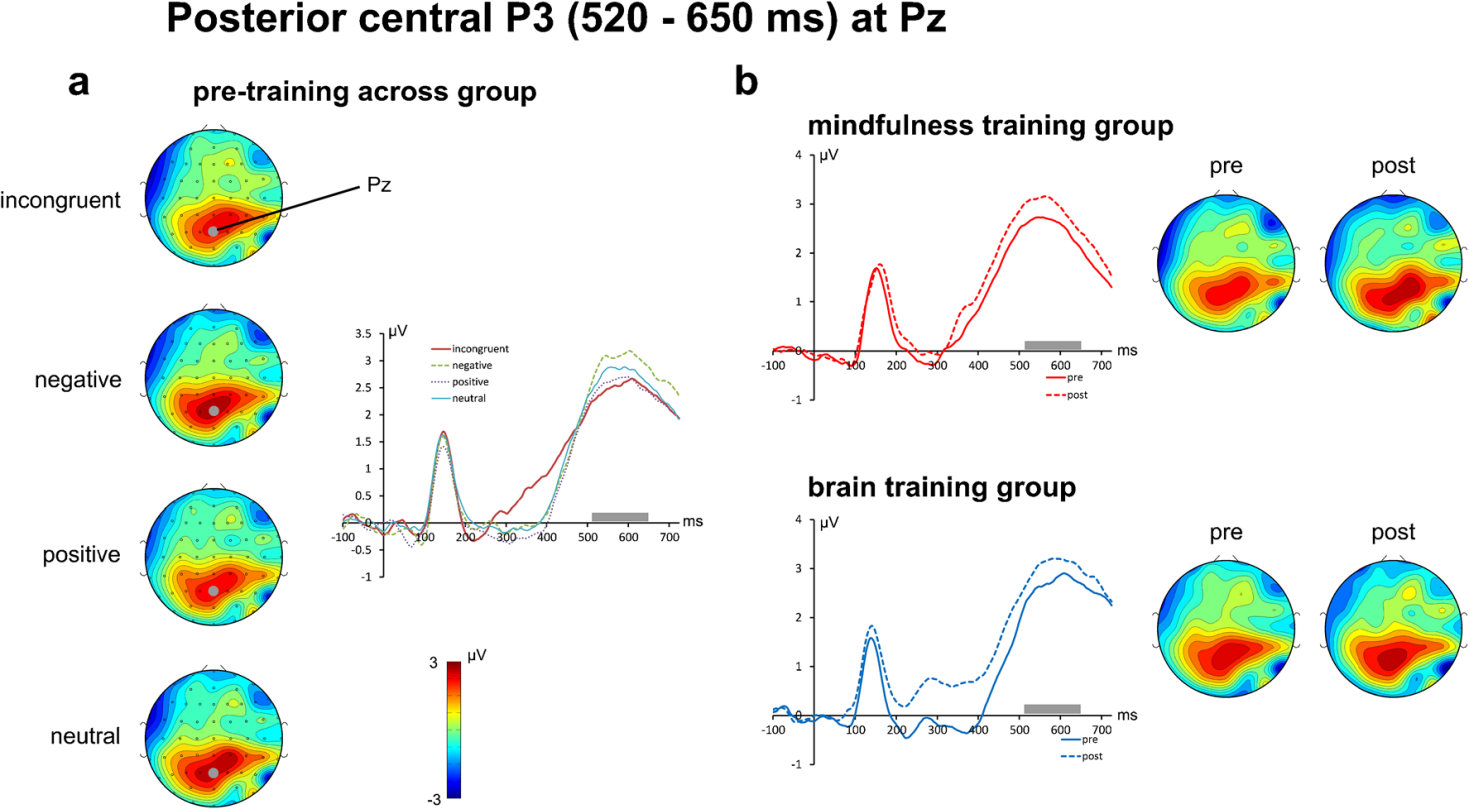
Event-related potentials (at electrode Pz) and spherical spline interpolated scalp topographies of the posterior central P3 potential. The *left panel* (**a**) sows the scalp topographies for T1 (pre-training) averaged across meditation and brain gym group. A clear P3 component captured in a time window from 520 to 650 ms post-stimulus (indicated by the *gray bars* in the ERP plots) and centered on electrode Pz is clearly visible. The *right panel* (**b**) shows the changes of the P3 from pre-training (*solid line*) to post-training (*dashed line*) for both groups

As shown in Fig. 5, the posterior P3b component was best captured by a time window from 520 to 650 ms post-stimulus at electrode Pz. A *Time* (2) × *Condition* (4) × *Group* (2) mixed ANOVA of this P3b mean amplitude only yielded a significant main effect of *Condition* [F(3,102) = 5.916, *p* = 0.001] with no other significant main or interaction effects (all *p* > 0.17). Post hoc analyses across groups and time points confirmed that the negative condition (3.18 μV) produced significantly higher P3b mean amplitudes than both the positive (2.63 μV) and incongruent (2.61 μV) conditions (both pairwise comparisons Bonferroni adjusted *p* < 0.01), but not for the neutral condition (2.86 μV), reflecting the same pattern of results as depicted in Fig. 5a for the pre-training data.

**Fig. 6:**
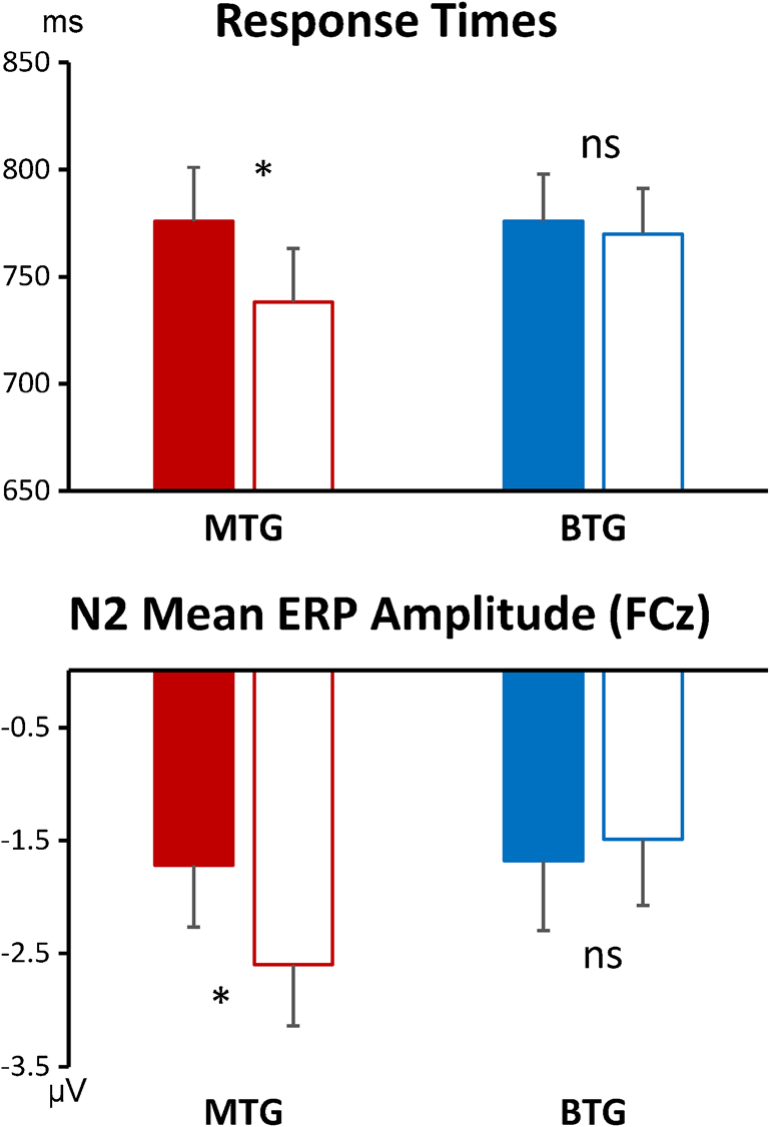
Summary of the main outcomes of the study, depicting improvements in response times and N2 ERP mean amplitudes for the meditation training group but not the brain training control group. The histograms represent mean response times and ERP amplitudes before and after training (*filled* and *unfilled bars*, respectively). *Error bars* indicate standard errors of the mean

Thus, the overall pattern of results shows that practice-induced changes only occured in the MTG, consisting of improvements in behavioral performance (RTs) and an increase of the N2 amplitude. Neither of these parameters improved in the BTG (see Fig. 6).

## Discussion

As hypothesized, positive behavioral and electrophysiological changes were observed after 55 to 75 year old adults engaged in mindful breath awareness practice, but not after engaging in a closely matched brain training program. The MTG improved RTs and increased fronto-central N2 mean amplitudes across conditions following 8 weeks of MT, whereas no significant changes were observed for the BTG. Of note, changes in fronto-central N2 mean amplitude, across groups, were significantly correlated with changes in RTs, with increases in N2 amplitudes related to faster responses. Thus, MT modulated neural resources of task-related stimulus processing, which directly contributed to more efficient behavioral responses.

Contrary to our expectations, MT did not specifically influence the processing of incongruent or of emotionally valenced stimuli. MT-related enhancements of the fronto-central N2 and improvements in RTs were seen across conditions, despite the fact that the congruency manipulation (reflected in slowed responses for incongruent stimuli) and the emotion manipulation (reflected in increased P3b mean amplitudes for negatively valenced stimuli) were successful. This pattern of results suggests that MT led to general improvements in task-related processing, rather than to a modulation of specific executive functions that are involved in conflict resolution and emotion regulation.

This interpretation is lent support by the fact that the observed N2 amplitude changes are primarily associated with the right angular gyrus (AG) and right superior parietal lobe, both part of the dorsal attention network which is involved in many aspects of attention regulation (Corbetta and Shulman 2011). More specifically, a range of neuroimaging studies using fMRI, EEG, and transcranial magnetic stimulation (TMS) link the right AG to the maintenance of goal-directed attention and to the updating (Rushworth and Taylor 2006) and dynamic allocation of attention to salient, task-relevant information (Ciaramelli et al. 2008; Arsalidou and Taylor 2011; Taylor et al. 2011; Seghier 2013; Singh-Curry and Husain 2009). Particularly pertinent to the visuospatial ecStroop task, Studer et al. (2014) demonstrated that decision making in such tasks is affected, when the AG is disrupted by TMS.

Based on the existing literature (Bekker et al. 2004, 2005; Liotti et al. 2000; Nieuwenhuis et al. 2003; van Veen and Carter 2002) we expected the ACC to be the main generator for the fronto-central N2 and meditation-specific changes to be related to it. Our source localization revealed that this was not the case. Although unexpected, the lack of modulation of the ACC and other frontal brain regions that are usually associated with executive functions (Raz and Buhle 2006), lends further support to the conclusion that MT did not improve executive control functions per se.

When considering the combination of meditation-related changes in our study, a clear interpretation emerges: The generally reduced response times, the increased N2 amplitudes across conditions, the localization of these changes in the right AG, and the lack thereof in the frontal brain areas converge onto an explanation that MT resulted in improvements of generic attentional processes, rather than conflict resolution or emotion regulation. In other words, the MTG group showed behavioral and neural indicators of improved maintenance of goal-directed attention in this visuospatial task.

A central question of this study was whether older participants (55–75 years of age) would benefit from MT and display improvements in their cognitive functions. The results of our study clearly confirm this and, to our knowledge, for the first time provide rigorously controlled evidence demonstrating improvements in cognitive performance as a result of simple mindfulness practice in this specific age group.

Because the MT-instructions included advice regarding a non-judgmental, non-reactive attentional stance toward arising thoughts and emotions it was expected that electrophysiological responses to valenced ecStroop stimuli would be selectively influenced by the mindfulness meditation training. However, this was not the case. No change in the processing of emotional stimuli for either group was observed. The P3b mean amplitude was larger for negatively than positively valenced stimuli across groups and time points. This generic effect indicates general valence-specific stimulus processing at stages that are usually associated with attentional control processes and the evaluation of semantic content. However, empirical evidence regarding the processing of affective words is rather varied and shows that the P3 and other late positive ERP components are affected by a whole range of variables, such as stimulus frequency, arousal levels, or processing demands (e.g., Briesemeister et al. 2014; Citron et al. 2013). As a result, so far, no consistent functional interpretation of the link between P3 modulations and the processing of valenced words has emerged in the literature (Citron 2012).

Irrespective of this gap in the literature, the general finding that the valence-sensitive P3 component did not change from T1 to T2 indicates that the processing of emotional stimuli was unaffected by MT. This result is not necessarily negative. In fact, this pattern of results is in line with some conceptualizations of MT which assert that focused attention needs to be trained first before a non-reactive and non-judgmental open monitoring state can successfully be cultivated (Lutz et al. 2008; Malinowski 2013b; Wallace and Shapiro 2006). This is to say, attentional improvements are likely to be seen *before* improvements in emotion regulation. From this perspective a possible explanation would be that the administered dose of MT was not sufficiently high to enhance emotional processing.

However, Allen et al. (2012) found that 6 weeks of MT resulted in positive improvements in attentional functions and concurrent improvements in the processing of affective stimuli with negative valence. Despite being 2 weeks shorter than the MT employed herein, the total amount of MT administered by Allen et al. was approximately 30 % greater than in the current study. Thus, additional MT experience might still be a plausible cause of the disparate results obtained by Allen et al. and those obtained herein. This conclusion is backed up by their finding that only those participants who engaged most in MT displayed reduced neural activation in response to negatively valenced stimuli.

A further difference to the study by Allen et al. (2012) was that the MT participants in their study engaged in additional practice aimed at developing empathy and compassion. This additional focus on emotional content may also account for the changes in emotional processing seen following short-term MT. Such additional instruction or counselling regarding emotion regulation are also typically used in mindfulness-based programs such as MBSR and MBCT and may provide crucial instruction for participants on how to process transient emotional states. Thus, the focus on breath awareness combined with only limited instructions how to regulate emotional processing may account for the lack of changes in emotional processing.

Alternatively, mindful breath awareness practices which include instructions regarding a non-judgmental, and accepting attitude, but are performed over a longer period or at a higher dosage and may produce improvements in emotional regulation without the need for additional instruction or counselling. Repeated practice would be expected to enable practitioners to interrupt pre-potent and automatic responses both inside and outside of the meditative state once the practitioner is able to consistently and successfully engage in mindfulness states that are characterized by a non-judgmental, accepting attitude and a non-reactive attentional stance. The amount of MT administered in the current study may simply have been too low a dose to observe such change.

A further explanation might be that the ecStroop paradigm does not sufficiently engage emotional processes. An effect of emotional words on behavioral performance is primarily observed when the emotional content is related to a particular affective dysfunction (J. M. G. Williams et al. 1996), whereas in general non-verbal emotional stimuli tend to elicit stronger neural effects than words (Citron 2012; Kissler et al. 2006).

In recent years, after completion of our data collection, concerns about using the FFMQ and similar questionnaires for tracking changes in trait mindfulness mounted. For example, the factor structure of the FFMQ is markedly different for meditators and non-meditators (M. J. Williams et al. 2014), suggesting that after introduction to mindfulness meditation participants may interpret the questionnaire items in different ways (see also Grossman and Van Dam 2011; Van Dam et al. 2009). Furthermore, Goldberg et al. (2015) contrasted mindfulness intervention and active control intervention and reported that the FFMQ did not differentiate between these groups. This lack of discriminant validity indicates that the FFMQ may be insensitive to mindfulness-specific changes and would explain why MT-specific improvements were only found for the *Observing* subscale and the curious decrease in *Acting with Awareness* in both groups.

In conclusion, the current study provides evidence that older participants, 55 to 75 years of age, who engaged in regular brief mindfulness training improved their performance in a cognitive task which requires visuospatial awareness and inhibitory control. Engaging in just over ten minutes of mindfulness practice five times per week over a period of 8 weeks resulted in significant improvements in behavioral and neurophysiological measures of general task-related processing. Employing a closely matched active control group and controlling for a broad range of further variables increases the confidence that these effects can be attributed to the mindfulness meditation practice. That the study did not find the expected specific improvements in executive control and in emotion regulation may be a result of the brevity of the intervention or may be due to the limited instructions to participants how to engage with their emotional experiences. Overall, the results are encouraging. Demonstrating that cognitive performance can be improved by a rather modest amount of mindfulness meditation practice encourages further explorations of MT as a potentially useful strategy for counteracting cognitive decline associated with aging. Although many questions are currently unanswered, the results demonstrate that further research into the potential preventative effects of mindfulness practice in terms of age-related declines is certainly promising.

## Electronic supplementary material

Below is the link to the electronic supplementary material.

ESM 1 (PDF 117 kb)

ESM 2 (PDF 304 kb)
