# Supplementary material for: Mindful Aging: The Effects of Regular Brief Mindfulness Practice on Electrophysiological Markers of Cognitive and Affective Processing in Older Adults
Source: Mindfulness (N Y). 2015 Dec 28;8(1):78–94. doi: 10.1007/s12671-015-0482-8 (PMC5241348; doi:10.1007/s12671-015-0482-8)
Supplement: Supplementary file 1 — (PDF 117 kb) [file 12671_2015_482_MOESM1_ESM.pdf]

Online Resource 1:

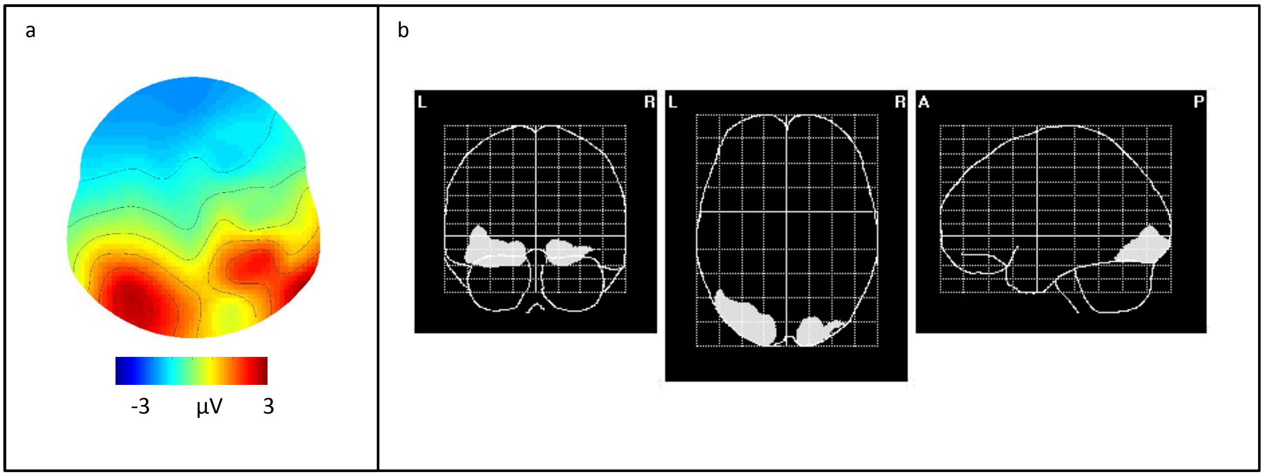

Panel a: Grand mean spherical-spline interpolated topographical map of the P1 ERP component (100-120 ms) averaged across all conditions. **Panel b:** Inverse solution of the P1 ERP component depicted in a glass brain. Statistically significant voxels are printed in white (Hotelling T^2 test against zero; $p < 0.05$). Centers of gravity: left and right lateral occipitotemporal gyrus (MNI coordinates: left X=-35 Y=-84 Z=-10, right X=21 Y=-91 Z=-13).

Mindful Aging: The effects of regular brief mindfulness practice on electrophysiological markers of cognitive and affective processing in older adults

Mindfulness

Peter Malinowski[1], Adam W. Moore[1] ,Bethan R. Mead[2], Thomas Gruber[3]

[1] Research Centre for Brain and Behaviour, Liverpool John Moores University
[2] Department of Psychological Sciences, Institute of Psychology, Health and Society, University of Liverpool
[3] Institute for Psychology, University of Osnabrück

✉ Peter Malinowski: p.malinowski@ljmu.ac.uk
