# Supplementary material for: Mindful Aging: The Effects of Regular Brief Mindfulness Practice on Electrophysiological Markers of Cognitive and Affective Processing in Older Adults
Source: Mindfulness (N Y). 2015 Dec 28;8(1):78–94. doi: 10.1007/s12671-015-0482-8 (PMC5241348; doi:10.1007/s12671-015-0482-8)
Supplement: Supplementary file 2 — (PDF 304 kb) [file 12671_2015_482_MOESM2_ESM.pdf]

Mindful Aging: The effects of regular brief mindfulness practice on electrophysiological markers of cognitive and affective processing in older adults

Mindfulness

Peter Malinowski[1], Adam W. Moore[1] ,Bethan R. Mead[2], Thomas Gruber[3]

[1] Research Centre for Brain and Behaviour, Liverpool John Moores University
[2] Department of Psychological Sciences, Institute of Psychology, Health and Society, University of Liverpool
[3] Institute for Psychology, University of Osnabrück

✉ Peter Malinowski: p.malinowski@ljmu.ac.uk

Tests for baseline differences

Supplementary Table 1 Summary of tests (independent t-tests; 2-tailed) for baseline differences of control variables, with mean values (standard deviations) and respective statistical values for the comparison between MTG and BTG

	**MTG (N=25)**	**BTG (N=25)**	**Statistical Values**	
Age (years)	65.3 (6.0)	64.0 (6.7)	$t(48) = .691$	$p = .493$
FFMQ-total	134.8 (17.6)	137.6 (18.6)	$t(48) = -.546$	$p = .587$
Computer Ability	6.0 (2.5)	6.4 (2.5)	$t(48) = -.644$	$p = .523$
Years in education (years)	13.2 (2.7)	13.3 (3.1)	$t(48) = -.147$	$p = .883$
Health**	8.0 (1.3)	7.6 (2.0)	$t(47) = .849$	$p = .400$
Speed of Processing (ms)	288 (46)	300 (46)	$t(48) = -.956$	$p = .344$
GSE	31.1 (4.2)	33.2 (4.0)	$t(48) = -1.830$	$p = .073$
WEMWBS	54.1 (7.0)	54.6 (9.8)	$t(48) = -.199$	$p = .843$
Cognitive Activity	42.8 (11.4)	50.6 (18.8)	$t(39.42^*) = -1.764$	$p = .084$
Physical Activity	32.5 (11.5)	33.6 (11.0)	$t(48) = -.351$	$p = .727$

* *Levene's test for equality of variances violated, therefore degrees of freedom were adjusted accordingly*
** *Only 24 participants from the MTG were included in this analysis due to missing data*

Supplementary Table 2 Summary of tests (independent t-tests; 2-tailed) for baseline differences of behavioural performance, with mean values (standard deviations) and respective statistical values for the comparison between MTG and BTG

	**MTG (n=22)**	**BTG (n=22)**	**Statistical Values**	
RT mean (ms)				
Incongruent	835 (140)	818 (120)	$t(42) = .437$	$p = .664$
Negative	762 (118)	766 (107)	$t(42) = -.141$	$p = .889$
Positive	759 (110)	761 (98)	$t(42) = -.084$	$p = .933$
Neutral	758 (117)	761 (96)	$t(42) = -.091$	$p = .928$
HR (%)				
Incongruent	92.5 (8.5)	96.5 (4.2)	$t(31^*) = -1.993$	$p = .055$
Negative	97.1 (2.6)	98.3 (2.2)	$t(42) = -1.674$	$p = .102$
Positive	97.7 (2.4)	98.6 (1.8)	$t(42) = -1.351$	$p = .184$
Neutral	97.4 (2.3)	97.9 (2.9)	$t(42) = -.625$	$p = .536$

* *Levene's test for equality of variances violated, therefore degrees of freedom were adjusted accordingly*

Tests for training-induced changes in control variables

Supplementary Table 3 Summary of FFMQ, GSE and WEMWBS pre and post mean values (standard deviations) and statistical values for the time x group interactions in the ANOVA

	**MTG (N=25)**		**BTG (N=25)**		**Statistical Values**	
	Pre	Post	Pre	Post	*F* (1,48)	*p*
FFMQ						
Non-reacting	22.7 (5.1)	22.6 (4.8)	22.3 (3.9)	22.2 (3.4)	0.002	.967
Observing	26.5 (5.6)	28.8 (4.3)	29.5 (4.4)	28.4 (3.9)	15.907	.001
Acting-aware	27.7 (5.5)	26.5 (5.8)	27.2 (5.3)	25.8 (5.1)	0.026	.872
Describing	28.6 (5.9)	30.4 (5.0)	30.6 (5.7)	30.6 (5.5)	3.343	.074
Non-judging	29.3 (6.2)	29.8 (6.1)	28.0 (7.3)	28.3 (6.6)	0.039	.844
Total	134.8 (17.6)	138.1 (16.6)	137.6 (18.6)	135.2 (15.1)	3.204	.080
GSE	31.1 (4.2)	32.2 (3.1)	33.2 (4.0)	34.1 (4.1)	0.081	.777
WEMWBS	54.1 (7.0)	55.2 (6.6)	54.6 (9.8)	54.3 (7.4)	0.889	.351
